# Cu-chitosan nano-net improves keeping quality of tomato by modulating physio-biochemical responses

**DOI:** 10.1038/s41598-020-78924-9

**Published:** 2020-12-14

**Authors:** Mahendra Meena, Shalini Pilania, Ajay Pal, Shiwani Mandhania, Bharat Bhushan, Satish Kumar, Gholamreza Gohari, Vinod Saharan

**Affiliations:** 1 Department of Horticulture, Rajasthan College of Agriculture, Maharana Pratap University of Agriculture and Technology, Udaipur, Rajasthan 313 001 India; 2 Department of Biochemistry, College of Basic Sciences and Humanities, Chaudhary Charan Singh Haryana Agricultural University, Hisar, Haryana 125 004 India; 3 ICAR-Indian Institute of Maize Research, PAU Campus, Ludhiana, Punjab 141 004 India; 4 ICAR-National Institute of Abiotic Stress Management, Baramati, Pune, Maharashtra 413 115 India; 5 Department of Horticulture, Faculty of Agriculture, University of Maragheh, 83111 - 55181 Maragheh, Iran; 6 Department of Molecular Biology and Biotechnology, Rajasthan College of Agriculture, Maharana Pratap University of Agriculture and Technology, Udaipur, Rajasthan 313001 India

**Keywords:** Nanoparticles, Plant physiology, Biochemical assays

## Abstract

Minimizing the post-harvest losses in fruits and vegetables is one of the challenging tasks in agriculture. To address this issue, we report nano-net of Cu-chitosan nanoparticles (Cu-chitosan NPs) which has the ability to extend the shelf-life of stored tomato. The application of Cu-chitosan NPs (0.01–0.04%) significantly curtailed microbial decay (< 5 versus > 50% in control), physiological loss in weight (14.36 versus 28.13% in control), respiration rate (0.01173 versus 0.01879 g CO_2_ kg^−1^ h^−1^) and maintained fruit firmness (34.0 versus 17.33 N in control) during storage. Further, these NPs significantly retarded loss of titratable acidity, retained total soluble solids, total and reducing sugars, lycopene, ascorbic acid and inhibited polyphenol oxidase. Likewise, NPs effectively preserved L^*^ (lightness), a* (red/green) and b* (blue/yellow) values and maintained organoleptic score. Scanning electron microscopy study confirmed that Cu-chitosan NPs orchestrate into an invisible-intangible nano-net over tomato surface which may plausibly act as a potential barrier at all possible openings (stem scar, cuticle wax, lenticels, and aquaporins) to control microbial infection, moisture loss, gas exchanges and respiration rate. Overall, nano-net extended keeping quality of tomatoes up to 21 days at room temperature (27 ± 2 °C, 55 ± 2% relative humidity).

## Introduction

Tomato (*Lycopersicon esculentum* L.) is one of the common vegetable crops, available throughout year and an economical source of minerals and antioxidants^1^. Ripened tomato is prone to physical injuries, microbial decay, declining quality, intolerable spoilage and thus incurs economical loss. Physical methods like post-harvest pre-cooling, refrigeration storage, post-harvest heat treatment, modified atmosphere packaging, etc. are common practices to minimize post-harvest losses in tomatoes. Such losses are predominantly common in developing countries because of the insufficient storage facility and poor transportation^1,2^. It has lead to overdoing of pre-/post-harvest application of agrochemicals like calcium chloride, 1-methylcyclopropene, nitric oxide, hydrogen peroxide, salicylic acid, sodium selenate, and fungicides to subside these losses^3^. Consumers at the same time are afraid of consuming chemical treated fruits and vegetables due to their unexpected health hazards^4–8^. Globally, post-harvest losses in tomato are 25–42% indicating the inadequacy of existing tools to reduce this loss and the need to develop robust measures which are not only economical to growers/traders but also safe for the consumers^1^. To deliver the next generation eco-friendly solutions, researchers have expedited work on diverse methods including nano-based products and processes to improve the keeping quality of climacteric fruits/vegetables like tomato^1^. Chitosan/nano-silica^9^, nano-Ca^10^, nano-TiO_2_^11^, limonene nano-coating^12^, nitric oxide-releasing chitosan nanoparticles^13^, thymol nanoemulsions + quinoa protein/chitosan edible films^14^, chitosan nanoparticles + *Byrsonima crassifolia* extract^15^, alginate + chitosan + ZnO nanoparticles^16^ and chitosan–surfactant nanostructure^6^ have been tried to extend the shelf-life of fruits and vegetables. Studies irrefutably indicate that chitosan has immensely been used as matrix for immobilizing/encapsulating the bioactive compounds to augment valuable functionalities like antimicrobial potential, physical barrier and antioxidant effect to extend the shelf-life of perishables. However, non-degradable highly reactive metal based nanomaterials are major concern for unexpected health hazards due to their residual effect in food. To provide safe solution to mentioned apprehensions, our research group has successfully developed various biodegradable chitosan based nanomaterials functionalized with Cu, Zn, and salicylic acid to prevent crop diseases and improve plant vigor^17–20^. Among these, Cu-chitosan NPs possess substantial antimicrobial and plant immune booster activities via up-regulating the antioxidant activity and maintaining the cellular homeostasis^17,18^. In these NPs, copper exists in its natural ionic form and is encapsulated into nano-scale chitosan matrix, and considered safer as compared to other zero valance, highly movable metallic nanomaterials. Additionally, the porous network architecture of Cu-chitosan NPs creates an antimicrobial ionic field through its entrapped Cu^17,18^. In spite of this, the composite effect of embedded Cu ions and chitosan is expected to be effective at low dose thus minimizing chemical load on treated fruits and vegetables. In view of aforesaid valuable bioactivities and unique properties of Cu-chitosan NPs, we hypothesized that these NPs would play a crucial role in reducing the post-harvest losses in fruits and vegetables. Therefore, in present study, Cu-chitosan NPs were applied on tomato fruits and concomitantly we report that NPs contrived a nano-net over tomato surface which persuaded intriguing responses over microbial decay, physiological loss in weight, and overall improved the keeping quality of tomatoes. The present study is first report of nano-net of Cu-chitosan NPs to improve the shelf-life of tomato up to 21 days at room temperature.

## Results

### Cu-chitosan NPs

**Figure 1:**
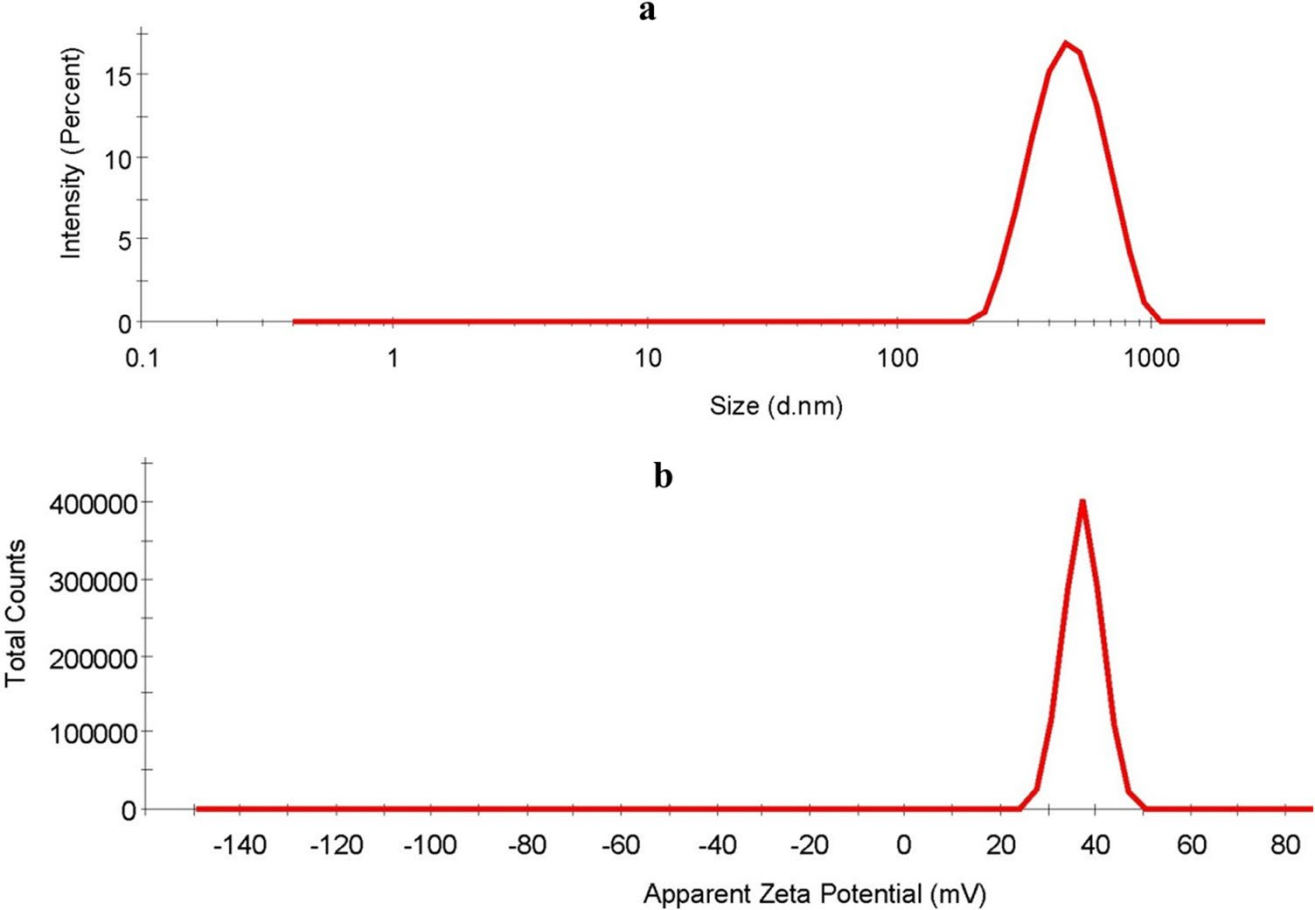
DLS analysis of Cu-chitosan NPs (**a**) size distribution by intensity and (**b**) zeta-potential.

DLS study showed similar physico-chemical characteristics viz*.* 368.3 ± 3.2 nm hydrodynamic diameter (Fig. 1a; see Supplementary Fig. S1 online), + 23.2 mV zeta-potential and 0.2 polydispersity index (PDI) (Fig. 1b; see Supplementary Fig. S1 online) of Cu-chitosan NPs as described earlier^17,18^. Cu-chitosan NPs have also been broadly studied for other physico-chemical properties by Fourier transform infrared spectroscopy (FT-IR), Transmission electron microscopy (TEM), Scanning electron microscopy (SEM) and elemental analysis by energy dispersive X-ray spectroscopy (EDX). FT-IR spectrum of Cu-chitosan NPs denoted shifting of peaks at 1636 cm^−1^ (CONH_2_) and 1550 cm^−1^ (NH_2_) to 1631 and 1536 cm^−1^ indicating the interactions of Cu with nano-scaled chitosan matrix (see Supplementary Fig. S1 online). TEM study described Cu accumulation into porous structured chitosan nanomaterials (see Supplementary Fig. S2 online). SEM–EDX analyses further confirmed the deposition of Cu into porous chitosan NPs (see Supplementary Fig. S3 online). Based on the physico-chemical characterization, a hypothetical synthesis and structural model of Cu-chitosan NPs was presented for better insights into Cu ions and chitosan interplay (see Supplementary Fig. S4 online)^17^.

### Effect of Cu-chitosan NPs on microbial decay of tomato

Microbial decay was visually inspected in stored tomatoes up to 21 days considering the extent of microbial infection on fruit surface in the scale ranging from 1 to 5. With the succession of storage period (0, 7, 14 and 21 d), microbes induced > 50% decay in control tomatoes while Cu-chitosan NPs (0.01 to 0.04%) remarkably inhibited microbial decay (5 to 0%) in stored tomatoes up to 21d (Table 1).

**Table 1:** Effect of Cu-chitosan NPs on % decay of tomato stored at room temperature.

Treatments (%)	Decay (%)			
	0 d	7 d	14 d	21 d
Control	0	20	50	> 50
CuSO_4_(0.01)	0	0	5	20
BCH (0.1)	0	5	20	50
**Cu-Chitosan NPs**				
0.01	0	0	0	5
0.04	0	0	0	0
0.08	0	0	5	20
0.12	0	0	5	20
0.16	0	5	20	50
0.20	0	5	20	50

Scale ranging from 1 to 5 were given to each treatment group, where 1 = normal (no decay on fruit surface), 2 = trace (up to 5% of fruit surface decayed), 3 = slight (5–20% of fruit surface decayed), 4 = moderate (20–50% of fruit surface decayed) and 5 = severe (> 50% of fruit surface decayed).Control with water. BCH (bulk-chitosan) dissolved in 0.1% acetic acid and CuSO_4_ (0.01%) dissolved in water.

### Effect of Cu-chitosan NPs on physiological parameters of tomato

**Figure 2:**
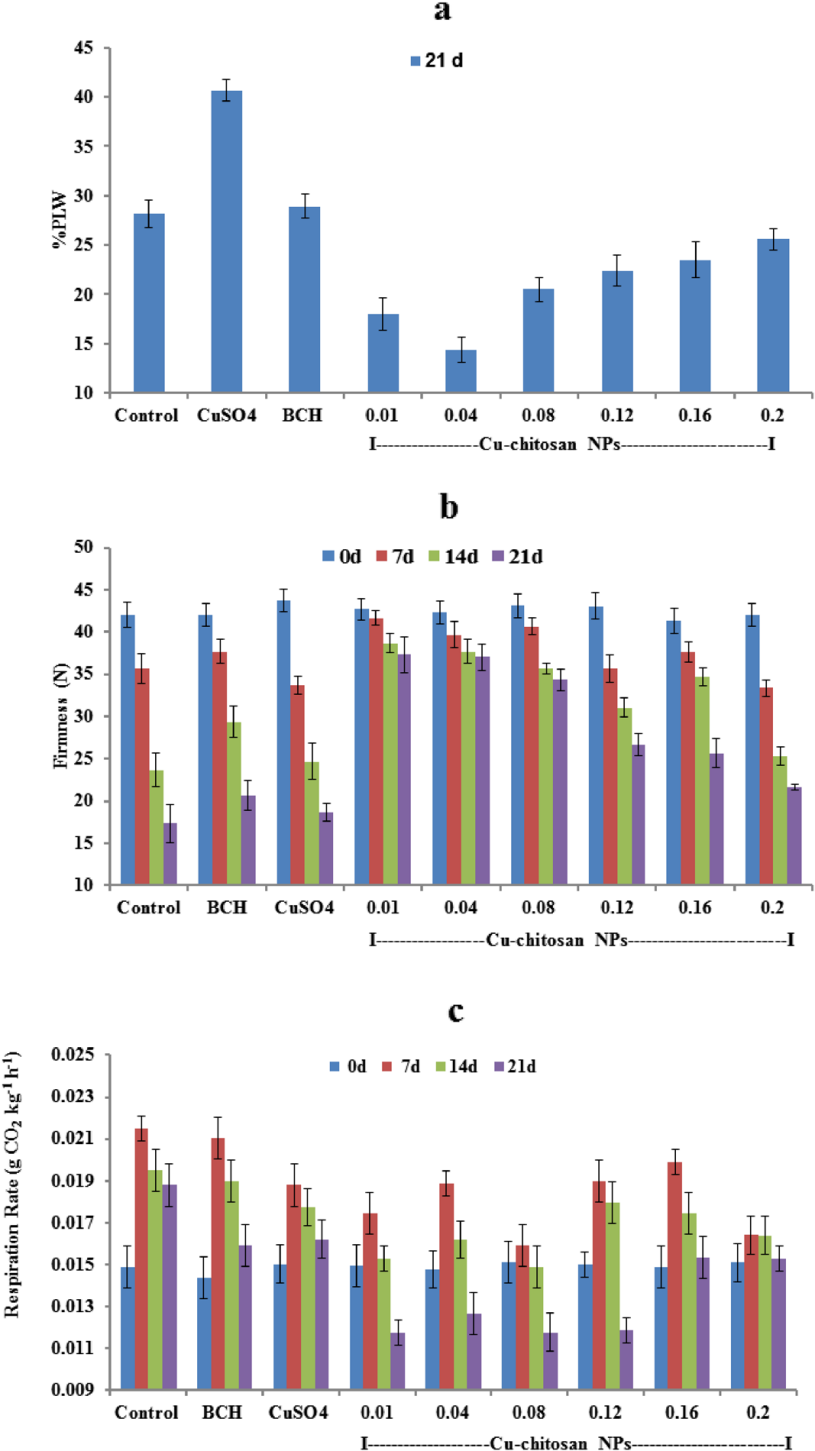
Effect of Cu-chitosan NPs on (**a**) PLW (**b**) firmness (**c**) respiration rate of tomato stored at room temperature. Each value is mean of triplicates and each replication consisted of 5 tomatoes. Control with water. BCH (bulk-chitosan) dissolved in 0.1% acetic acid and CuSO_4_ (0.01%) dissolved in water. Error bars represents ± SE (standard error).

Physiological loss in weight (PLW, %), firmness (N), and respiration rate (g CO_2_ kg^−1^ h^−1^) were measured at 0, 7, 14, and 21 d of storage at room temperature. Maximum PLW (28.13%) was recorded in control tomatoes at 21 d of storage. In similar storage period, Cu-chitosan NPs (0.04%) considerably controlled PLW and exhibited only 14.36% weight loss (Fig. 2a). In control tomato, fruit firmness decreased from 43.0 to 17.3 N from 0 to 21d of storage while it was significantly maintained in tomatoes treated with 0.01 to 0.12% Cu-chitosan NPs (Fig. 2b). Similarly, tomatoes treated with Cu-chitosan NPs (0.1 to 0.12%) showed significant control on respiration rate (0.01173 to 0.01266 g CO_2_ kg^−1^ h^−1^) as compared with control (0.01879 g CO_2_ kg^−1^ h^−1^) (Fig. 2c).

### Effect of Cu-chitosan NPs on quality attributes of tomato

**Figure 3:**
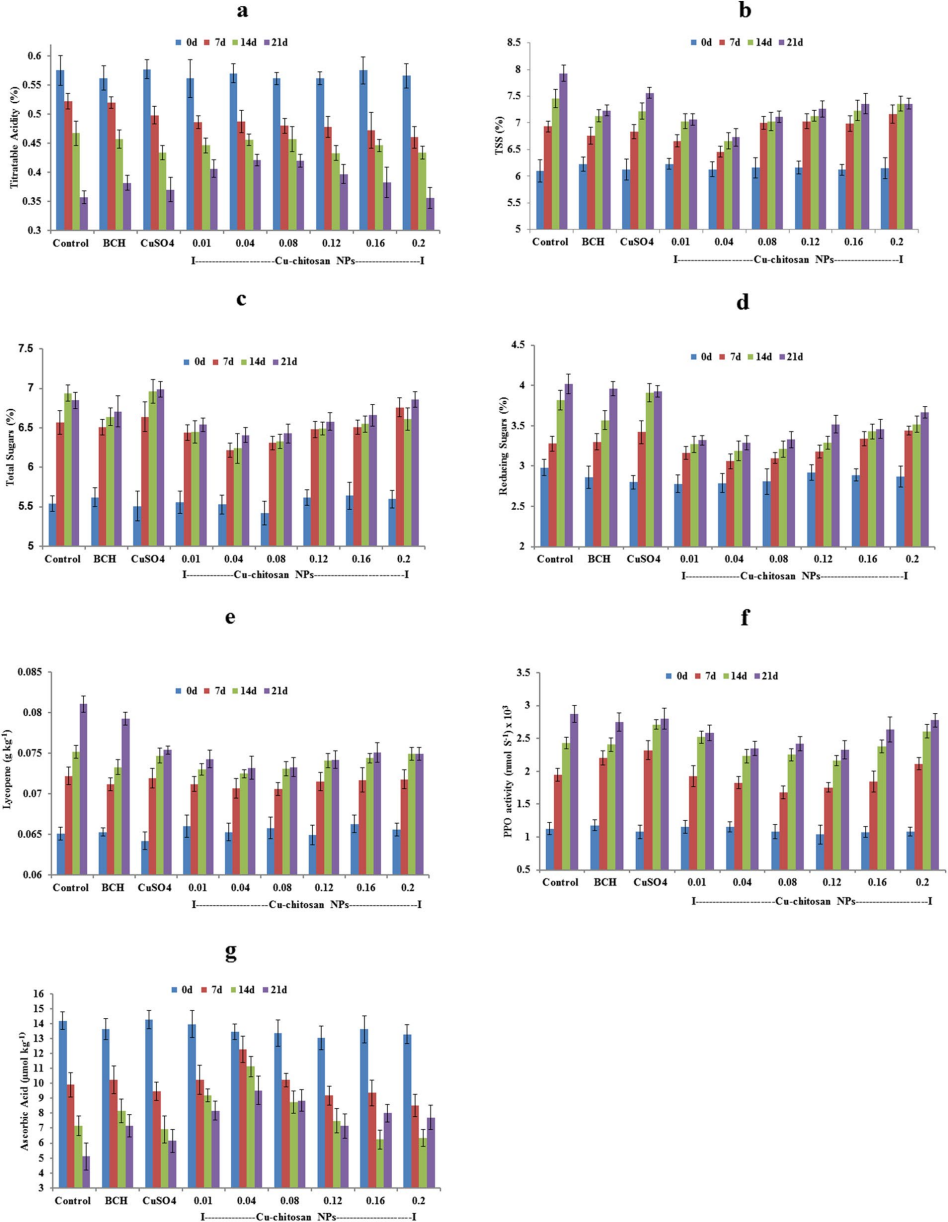
Effect of Cu-chitosan NPs on (**a**) titratable acidity (**b**) total soluble solids and (**c**) total sugar (**d**) reducing sugars (**e**) lycopene (**f**) polyphenol oxidase and (**g**) ascorbic acid in tomato stored at room temperature. Each value is mean of triplicates and each replication consisted of 5 tomatoes. BCH (bulk-chitosan) dissolved in 0.1% acetic acid and CuSO_4_ (0.01%) dissolved in water. Error bars represents ± SE (standard error).

Titratable acidity (TA) of tomato declined throughout storage period in all the stored tomatoes. The decline was lesser in tomatoes treated with Cu-chitosan NPs (0.01–0.16%) as compared with control from 14 to 21d of storage (Fig. 3a). Tomatoes treated with Cu-chitosan NPs considerably abated the increase of total soluble solids (TSS), total and reducing sugars, lycopene, and PPO activity as compared with control (Fig. 3b–f). Likewise, tomatoes treated with Cu-chitosan NPs significantly retained ascorbic acid (0.16 g kg^−1^) as compared with control (0.090 g kg^−1^) **(Fig.** **3****g**).

### Scanning electron microscopy (SEM) of tomato surface

**Figure 4:**
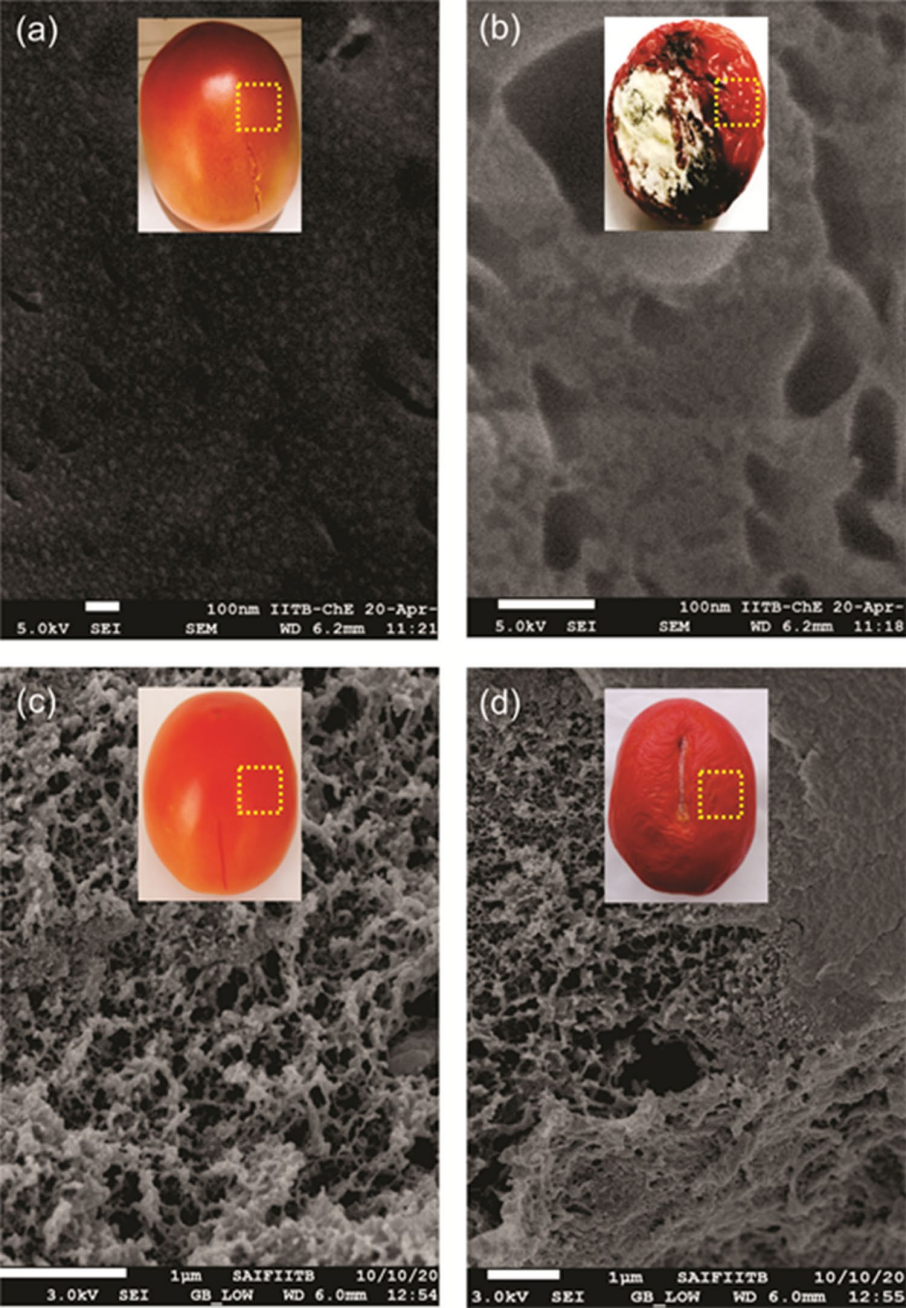
SEM study of tomato surface (**a**) control tomato at day 1 and (**b**) day 21; Cu-chitosan NPs treated tomato at (**c**) 1 and (**d**) 21 d.

At first day, the surface of control tomato exhibited scattered lenticels and at 21 d of storage, tomato got decayed and displayed large fissures on cuticle wax surface (Fig. 4a,b). In contrast, at 1 and 21 d, Cu-chitosan NPs (0.04%) treated tomato showed nano-net structures over the tomato surface (Fig. 4c,d).

### Effect of Cu-chitosan NPs on colour attributes, organoleptic score and Cu content in tomato

**Figure 5:**
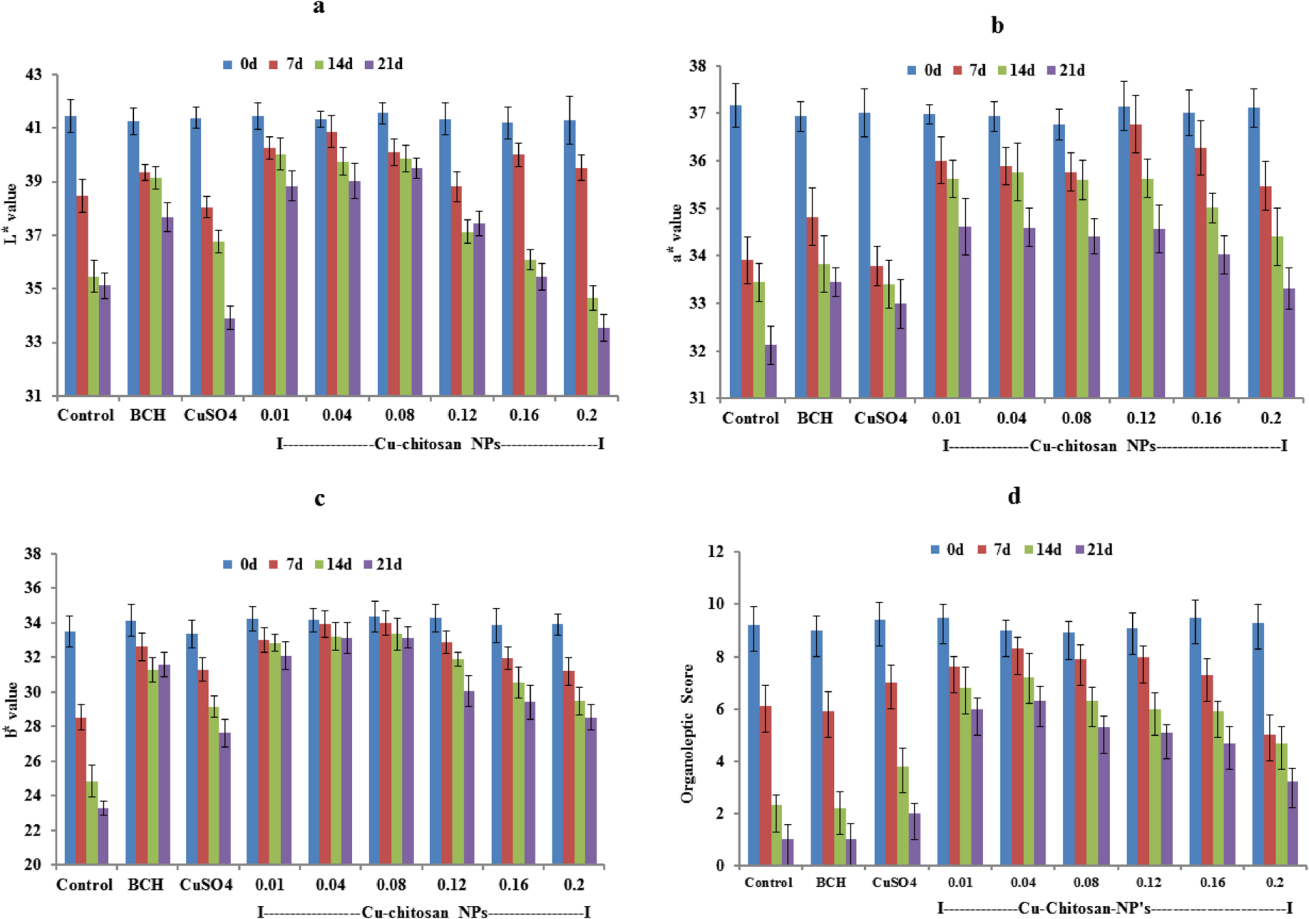
Effect of Cu-chitosan NPs on (**a**) L٭value (**b**) a٭value (**c**) b٭value and (**d**) organoleptic score in tomato stored at room temperature. Each value is mean of triplicates and each replication consisted of 5 tomatoes. BCH (bulk-chitosan) dissolved in 0.1% acetic acid and CuSO_4_ (0.01%) dissolved in water. Error bars represents ± SE (standard error).

The colour values (L^*^, a^*^, b^*^) decreased during storage period in control tomatoes while the same were notably maintained in Cu-chitosan NPs treated tomatoes during storage (Fig. 5a–c). Organoleptic scores, given on a five-point hedonic scale (9-Excellent, 7-Very good, 5-Good, 3-Fair, 1-Poor), were > 4 in Cu-chitosan NPs treated tomatoes up to 21 days of storage (Fig. 5d). After 21 days of storage, a non-significant change in Cu^+2^ (0.028–0.035 g kg^−1^ pulp) was observed in Cu-chitosan NPs (0.01 to 0.08%) treated tomatoes as compared with control (0.027 g kg^−1^ pulp). However, the maximum accumulation of Cu^+2^ (0.060 g kg^−1^) was recorded in tomatoes treated with CuSO_4_ after 21d of storage (Table 2).

**Table 2:** Effect of Cu-chitosan NPs on Cu^+2^ content in tomato. Data were recorded in tomato after 21 d of storage.

Treatment (%)	Cu^+2^ (g kg^−1^)
Control (water)	0.027 ± 0.02^ g^
BCH (0.1)	0.026 ± 0.11^ g^
CuSO_4_ (0.01)	0.060 ± 0.13^a^
**Cu-chitosan NPs**	
0.01	0.028 ± 0.04^ g^
0.04	0.031 ± 0.12f.
0.08	0.035 ± 0.17^e^
0.12	0.045 ± 0.10^d^
0.16	0.049 ± 0.20^c^
0.20	0.058 ± 0.10^b^

Each value is mean of triplicates and each replication consisted of 5 tomatoes. Mean ± SE followed by same letter is not significantly different at *p* = 0.05 as determined by Tukey − Kramer HSD. BCH represents bulk chitosan (BCH) dissolved in 0.1% acetic acid and CuS0_4_ dissolved in water.

## Discussion

chitosan–surfactant nanostructure, alginate + chitosan + ZnO nanoparticles, limonene nano, active packaging of chitosan + TiO_2_ NPs, nitric oxide-releasing chitosan nanoparticles, nano-Ca, thymol nanoemulsion + quinoa protein/chitosan edible films and chitosan + nano-silica have been evaluated in tomato, loquat, strawberry, apple, sweet cherry, cherry tomato, black grape, and guavas to study post-harvest losses^6,9–14,16^. The literature survey indicates that chitosan has predominantly been preferred material to minimize post-harvest losses in perishables due to its non-toxic, biodegradable, film-forming, and edible nature^21^. Various chitosan nanomaterials functionalized with Cu, Zn, salicylic acid, etc. have established their role in endorsing plant cellular homeostasis through up-regulation of antioxidant-defense enzymes to protect crops from diseases^17–20,22^. Amongst these chitosan nanomaterials, Cu-chitosan NPs are known to inhibit various plant pathogenic microbes and incite plant defense-antioxidant system for broad-spectrum disease resistance^17,18,22^. Additionally, We envisage that various favorable physico-chemical characteristics viz*.* size (368.3 ± 3.2 nm), mono-dispersity (0.2 PDI), positive zeta-potential (+ 22 mV) and porous architecture of Cu-chitosan NPs provided it add-on features to facilitate uniform and firm binding to cuticular wax surfaces of fruits and vegetables (Fig. 1a,b; see Supplementary Figs. S2, S3). Further, encapsulated Cu ions into nano-scaled chitosan matrix endowed robust antimicrobial activity to Cu-chitosan NPs (see Supplementary Fig. S4). In this realm, we hypothesized that Cu-chitosan NPs would irrefutably be effective to maneuver shelf-life of fruits and vegetables. Therefore, we evaluated Cu-chitosan NPs for the first time to study various factors concomitant to post-harvest losses in tomato.

**Figure 6:**
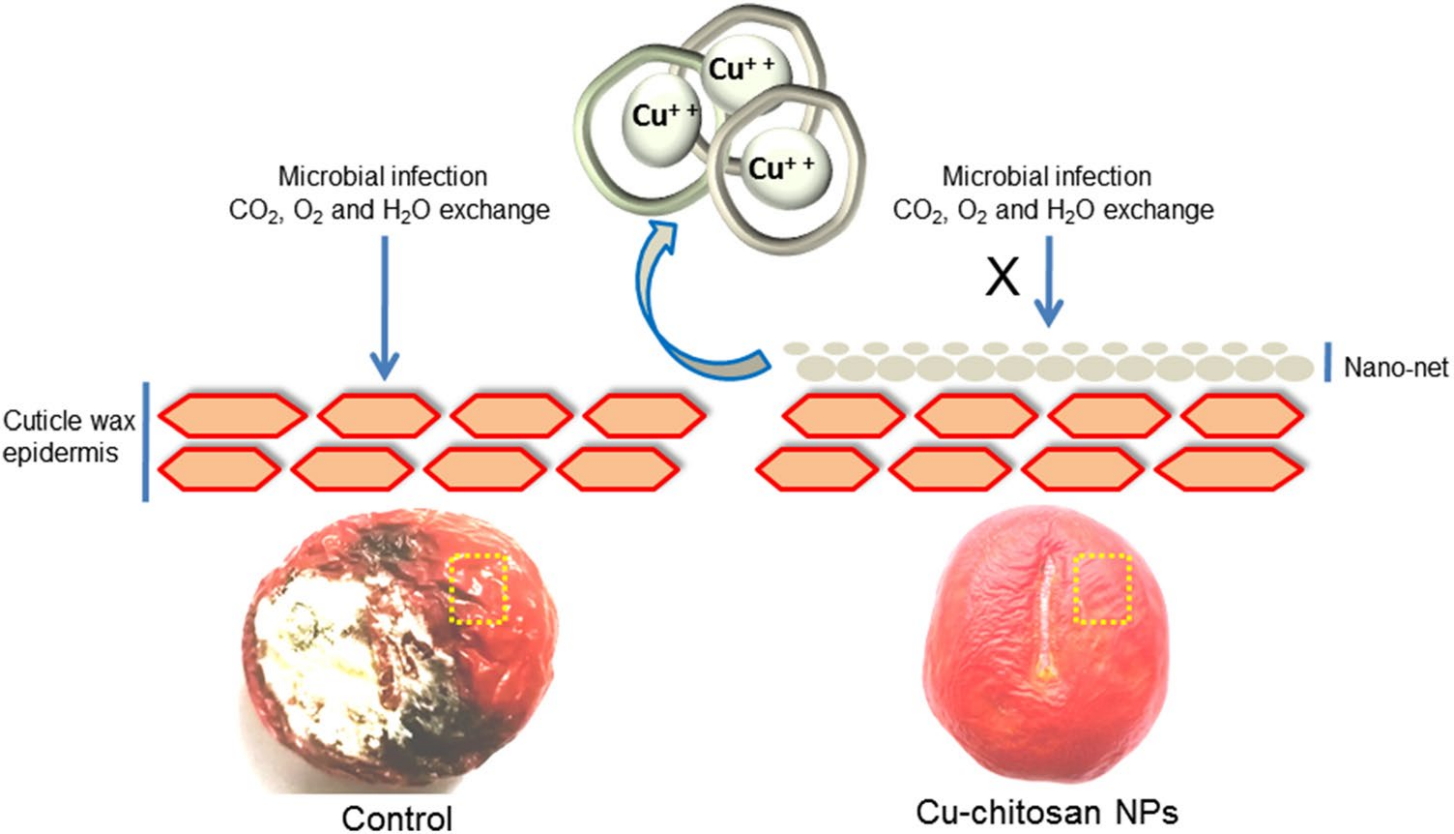
Hypothetical model of nano-net on tomatoes to extend the self-life.

Curbing microbial infection is of utmost importance as it causes direct rotting and aggravates physiological disorders in stored fruits and vegetables. In the present study, Cu-chitosan NPs significantly precluded microbial infection and notably reduced decay in stored tomatoes (Table 1). Elusive bioactivity of Cu-chitosan NPs on microbial decay was not only due to antimicrobial nature of chitosan and Cu^+2^ but also by their effect on preventing plant cell death by enhancing plant cell immunity^17^. Moreover, a severe microbial infection on the tomato surface might have created a local acidic environment to induce the quick release of Cu^+2^ from the chitosan matrix to swiftly inhibit microbial infectivity^18^. Altogether, we assume that reported nano-net of Cu-chitosan NPs over the tomato surface acts as a nano-shield and elegantly prevents the microbial infection at low concentrations (0.01–0.04%) as compared to earlier reports (Figs. 4c,d; 6).

Further, Cu-chitosan NPs comprehensively ameliorated various physiological disorders by significantly abating PLW, maintaining fruit firmness, and decreasing respiration rate in stored tomatoes (Fig. 2a–c). Higher respiration rate in fruit induces metabolic activities, speeds up ripening, and decreases the shelf-life of tomatoes. In our findings, Cu-chitosan NPs significantly controlled respiration rate during storage (Fig. 2c). Earlier, TiO_2_ embedded chitosan nanocomposite film was found to retard respiration rate and retain firmness in cherry tomato up to 15 d of storage at 20 °C^11^. In fruits and vegetables, gas exchange and moisture loss and even microbial entry mainly occur through stem scar, cuticular wax, lenticels, and aquaporins^23–25^. In present investigation, SEM study confirmed that Cu-chitosan NPs builds a nano-net over tomato surface and we assume that it acts as a potential barrier at all possible openings (stem scar, cuticle wax, lenticels, and aquaporins) to control microbial infection, moisture loss, gas exchanges/ respiration rate (Figs. 4c,d; 6). Contrarily, severe fissures were evident on the cuticular wax surface of control samples after 21 d of storage which were responsible for faster decay of tomatoes (Fig. 4b). These large fissures on tomato surface were caused by severe microbial infection and shrinking of tomato due to unrestricted moisture loss and gas exchange. The sour taste of tomato is largely due to its TA which is considered a key quality parameter of tomato. TA in tomato decreased during storage, whereas the application of Cu-chitosan NPs significantly conserved it (Fig. 3a). We foresee that chitosan of Cu-chitosan NPs decreased respiration through nano-net and the low respiration rate eventually averts the consumption of organic acids^26^. During storage, TSS increased from 6.1 to 7.93% from 1 to 21 d in untreated tomatoes, while Cu-chitosan NPs (0.01 to 0.20%) significantly hold it between 7.06 to 6.46% in 21 d of storage as compared to an earlier report by Ali and co-workers (Fig. 3b)^27^. Based on these findings, we further anticipate that chitosan at the nano-scale has higher potential to precisely control tomato TSS during storage. During ripening, starch degrades to yield glucose which contributes to the sweetness of fruit. Therefore, to extend the shelf-life of fruit, contents of total and reducing sugars needs to contain and Cu-chitosan NPs significantly controlled sugars during storage (Fig. 3c,d). The observed results could be attributed to the fact that Cu-chitosan NPs considerably suppress respiration and metabolic activities which in turn control total and reducing sugars. Color change in tomato during storage occurs due to the buildup of lycopene into chromoplasts and this process speeds up with respiration^28^. Cu-chitosan NPs considerably controlled respiration rate thus abated lycopene synthesis during storage (Fig. 3e). Higher activity of PPO is an indicator of post-harvest losses as it is responsible for changes in colour, flavor, and loss of nutritional value. Cu-chitosan NPs remarkably inhibited its activity in tomato during storage as compared with control (Fig. 3f). Ascorbic acid is one of the key antioxidant compounds present in high concentration in green tomato and decreases during ripening^29^. In the present study, ascorbic acid severely decreased from 0.25 to 0.09 g kg^−1^ in 21 days of storage in control fruits (Fig. 3g). Cu-chitosan NPs, however, significantly maintained the level of ascorbic acid throughout storage period as compared to earlier report on cherry tomato stored in active packaging of chitosan TiO_2_ nano-composites^11^. L^*^ (lightness), a* (red/green) and b* (blue/yellow) values and organoleptic score (sensory evaluation) are important parameters that affect the acceptability of fruits in the market. Tomatoes treated with Cu-chitosan NPs significantly conserved L^*^, a^*^ and b^*^ values and yielded fair to good organoleptic score during storage (Fig. 5a–d).

AAS studies were conducted to assess the Cu^+2^ content in stored tomatoes. As compared to control, a non-significant increase in Cu^+2^ content (0.028–0.035 g kg^−1^ pulp) was found in tomatoes treated with Cu-chitosan NPs (0.01–0.08%) which is below the toxic level^30^. However, the maximum accumulation of Cu^+2^ (0.060 g kg^−1^) was recorded in tomatoes treated with CuSO_4_. These results strongly point out that Cu-chitosan NPs of 368 nm diameter exists as a net structure and hardly infiltrates through the possible openings viz. stem scar, cuticular wax, lenticels and aquaporins into tomato. Majority of nanomaterials used *in planta* are zero valency metal NPs and below 100 nm size, thus have higher probability of infiltration and translocation into living organisms and raise serious apprehensions^31–34^. Nano-based materials that are directly or indirectly used in food materials especially for coatings and packaging of fruit and vegetables need to be biodegradable and biocompatible. Herein, we claim that myriad and fecund response of Cu-chitosan NPs to improve the keeping quality of tomato has potential to expend to other fruits and vegetables.

## Conclusion

Minimizing post-harvest losses in fruits and vegetables with safe, effective and economical means is a major challenge in agriculture. Demand of chemical free food has unexpectedly risen due to increasing hazardous effects of chemicals on consumers. In this line, Cu-chitosan nanomaterials which are effective at low dose, biodegradable and easy to use, could be the fair alternative of hazardous chemicals. Thus all together, developed nanoformulation has potential to be translated into valuable technology to enhance the shelf-life and quality of fruits and vegetables during prolonged storage.

## Methods

### Materials

Chitosan (80% N-deacetylation, Mol. Wt. 50,000–190,000 Da) and sodium tri-polyphosphate (TPP) were procured from Sigma-Aldrich, St. Louis, MO, USA. Chemicals and key reagents for biochemical assays were procured from HiMedia and SRL, Mumbai, India. Tomato variety *Dev*, grown at the university farm of Maharana Pratap University of Agriculture and Technology, Udaipur, India was used in the present study.

### Cu-chitosan NPs

Dry powder of Cu-chitosan NPs was prepared by method described in our earlier study^20^. Before use, NPs were characterized for mean hydrodynamic diameter, zeta-potential, and polydispersity index (PDI) by dynamic light scattering (DLS) using Zetasizer ZS 90 (Malvern, UK) at 25 °C at a scattering angle of 90° in triplicate.

### Treatment of Cu-chitosan NPs on tomato

Stem detached tomatoes of similar size at the orange stage were selected and mechanically damaged with visible decay were abandoned for the study. Selected tomatoes were dipped into different concentrations of Cu-chitosan NPs (0.01, 0.04, 0.08, 0.12, 0.16 and 0.20%, w/v in water), bulk-chitosan (BCH) (0.1%), CuSO_4_ (0.01%, w/v) and water (control) for 6 min, and kept in well ventilated paper baskets for storage at room temperature (27 °C ± 2) with the relative humidity 55 ± 2%.

### Microbial decay

Before treatments, 1.0 cm long and 0.5 cm deep mechanical cut was placed on tomatoes for induction of microbial infection. Microbial decay was visually inspected in each treatment considering the extent of fungal growth on tomato surface in the scale ranging from 1 to 5, where 1 = normal (no decay on fruit surface), 2 = trace (up to 5% of fruit surface decayed), 3 = slight (5–20% of fruit surface decayed), 4 = moderate (20–50% of fruit surface decayed) and 5 = severe (> 50% of fruit surface decayed)^35^.

### Physiological loss in weight (PLW), firmness, and respiration rate

Initial weights of tomatoes were recorded before (0 d) and after treatment (7, 14 and 21 d). Weight loss was recorded as the difference between initial and final weight (taken on a particular day) and expressed as % PLW^36^. The firmness was determined using Texture Analyzer (Model TA- XT Plus, Stable micro System Limited, Godalming, Surrey, UK) equipped with a cylinder probe of 50 kg load cell. Indirect respiration rate (g CO_2_ kg^−1^ h^−1^) in a static system was determined following the method described earlier^37^. In brief, samples (~ 150 g) were placed in polythene bags with silicon septum and CO_2_ concentration was measured using HeadSpace Gas Analyzer (WITT- GASETECHNIK GmbH & Co KG, Witten, Germany). After 24 h, CO_2_ concentration was again measured and the difference in CO_2_ concentration was calculated.

### Titratable acidity (TA) and total soluble solids (TSS)

TA (%) of tomato juice was determined by titrating a known volume of diluted tomato juice against standard N/10 NaOH solution using phenolphthalein as an indicator until a faint pink color appeared. The results were expressed as percent acidity of the fruit juice^38^.Total soluble solids (%) in the juice were determined using “Pocket Refractometer” bearing the range 0–53%^38^.

### Total sugars, reducing sugars, lycopene, ascorbic acid and polyphenol oxidase (PPO)

Total sugars (%), reducing sugars (%) and lycopene (g kg^−1^) contents were measured using anthrone, dinitrosalicylic acid and anhydrous sodium sulphate methods, respectively using spectrophotometer^38^. PPO activity (nmol s^−1^) was measured using a spectrophotometer following the method described by Taneja and Sachar^39^. Ascorbic acid was determined by diluting a known volume of filtered juice with 3% metaphosphoric acid and titrating against 2, 6- dichloro phenol indophenol till a stable light pink color appeared. The results were expressed as µmol kg^−1^^38^.

### Scanning electron microscopy (SEM) of the tomato surface

To study the tomato surface, SEM analyses were performed in epidermis samples of treated and control tomatoes (at 1 and 21 d of storage). Samples were freeze-dried for 8 h and mounted on double-sided carbon aluminum stubs and sputter-coated with gold–palladium (SC7620, Emitech). SEM images were taken in high vacuum mode using Zeiss EVO MA10 SEM (Carl Zeiss Promenade, Germany) at 20 kV.

### Hunter colour values and overall organoleptic score

Changes in L^*^, a^*^, b^*^ colour coordinates on the Hunter scale were measured using a colorimeter (Chroma Meter CR 400; Konica Minolta, Tokyo, Japan). The color measurements were expressed in terms of luminosity (lightness) L^*^ (L^*^ = 0 for black and L^*^ = 100 for white) and the chromaticity parameters a (green [-] to red [ +]) and b (blue [-] to yellow [ +]). Overall acceptability of samples was evaluated through standard sensory evaluation techniques. Sensory attributes such as taste, flavor and acceptability were rated on a five point hedonic scale (9-Excellent, 7-Very good, 5-Good, 3-Fair, 1-Poor) by a selected panel of judges (11 members).

### Estimation of Cu^+2^ content

Treated and control tomatoes after 21d of storage were washed properly and pulp samples were taken for estimation of Cu^+2^. Samples were dried in oven at 80ºC, burnt in muffle oven and white ash was dissolved in 6 N HCl. Cu^+2^ content was measured using the atomic absorption spectrophotometer (AAS 4141 model, Electronics Corp. of India Ltd., India)^40^.

### Statistical analysis

Statistical analysis was performed using JMP software version 12^41^ using Turkey Kramer HSD test. Each experiment was repeated twice wherein each treatment consisted of minimum of three replicates with five tomatoes in each replication.

### Supplementary information

Supporting data include physico-chemical characteristic of Cu-Chitosan NPs viz*.* size distribution, zeta-potential, FTIR spectra (Fig. S1); external-internal architecture, elemental analysis (Fig. S2) and hypothetical model (Fig. S4).

## Supplementary information

Supplementary Information.
