# Supplementary material for: Cu-chitosan nano-net improves keeping quality of tomato by modulating physio-biochemical responses
Source: Sci Rep. 2020 Dec 14;10:21914. doi: 10.1038/s41598-020-78924-9 (PMC8097068; doi:10.1038/s41598-020-78924-9)
Supplement: Supplementary file 1 — Supplementary Information. [file 41598_2020_78924_MOESM1_ESM.pdf]

Supplementary Data

Cu-chitosan nano-net improves keeping quality of tomato by modulating physio-biochemical responses

Mahendra Meena[1], Shalini Pilania[1], Ajay Pal[2], Shiwani Mandhania[2], Bharat Bhushan[3], Satish Kumar[4], Gholamreza Gohari[5] and Vinod Saharan[6]*

[1]Department of Horticulture, Rajasthan College of Agriculture, Maharana Pratap University of Agriculture and Technology, Udaipur, Rajasthan 313 001, India

[2]Department of Biochemistry, College of Basic Sciences and Humanities, Chaudhary Charan Singh Haryana Agricultural University, Hisar, Haryana 125 004, India

[3]ICAR-Indian Institute of Maize Research, PAU Campus, Ludhiana, Punjab 141 004, India

[4]ICAR-National Institute of Abiotic Stress Management, Baramati, Pune, Maharashtra 413 115, India

[5]Department of Horticulture, Faculty of Agriculture, University of Maragheh, Maragheh 83111 - 55181, Iran

[6]Department of Molecular Biology and Biotechnology, Rajasthan College of Agriculture, Maharana Pratap University of Agriculture and Technology, Udaipur, Rajasthan 313001, India

***Corresponding author**

E-mail: vinodsaharan@gmail.com

Phone: +91-9461180586; Fax: +91-294-2420447

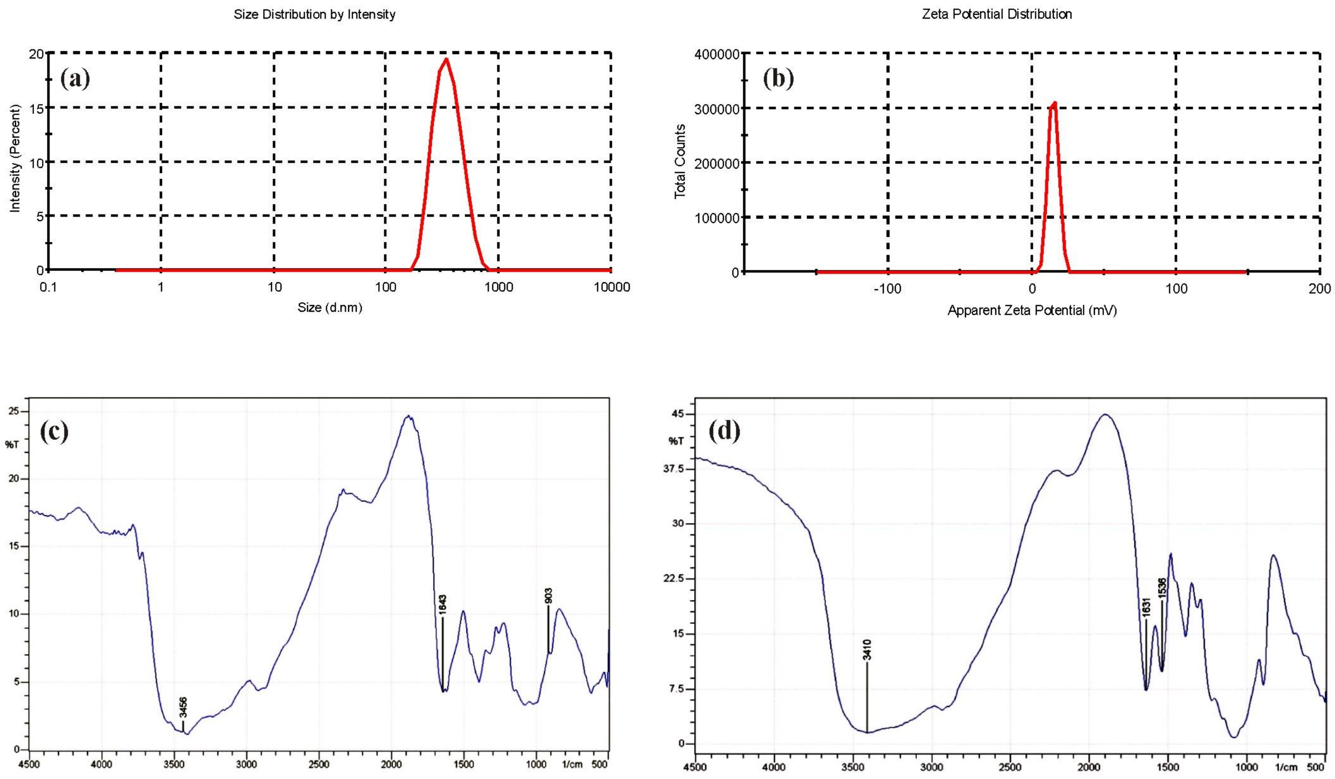

Figure S1. DLS analysis of Cu–chitosan nanoparticles (a) size distribution by intensity, and (b) zeta potential distribution. FTIR spectra (c) Bulk chitosan, and (d) Cu–chitosan nanoparticles. X-axis of (c) and (d) is wave number (cm^{-1}). *The figure is adopted from reference (Saharan et al 2015)* [17]*with copyright permission from Elsevier.*

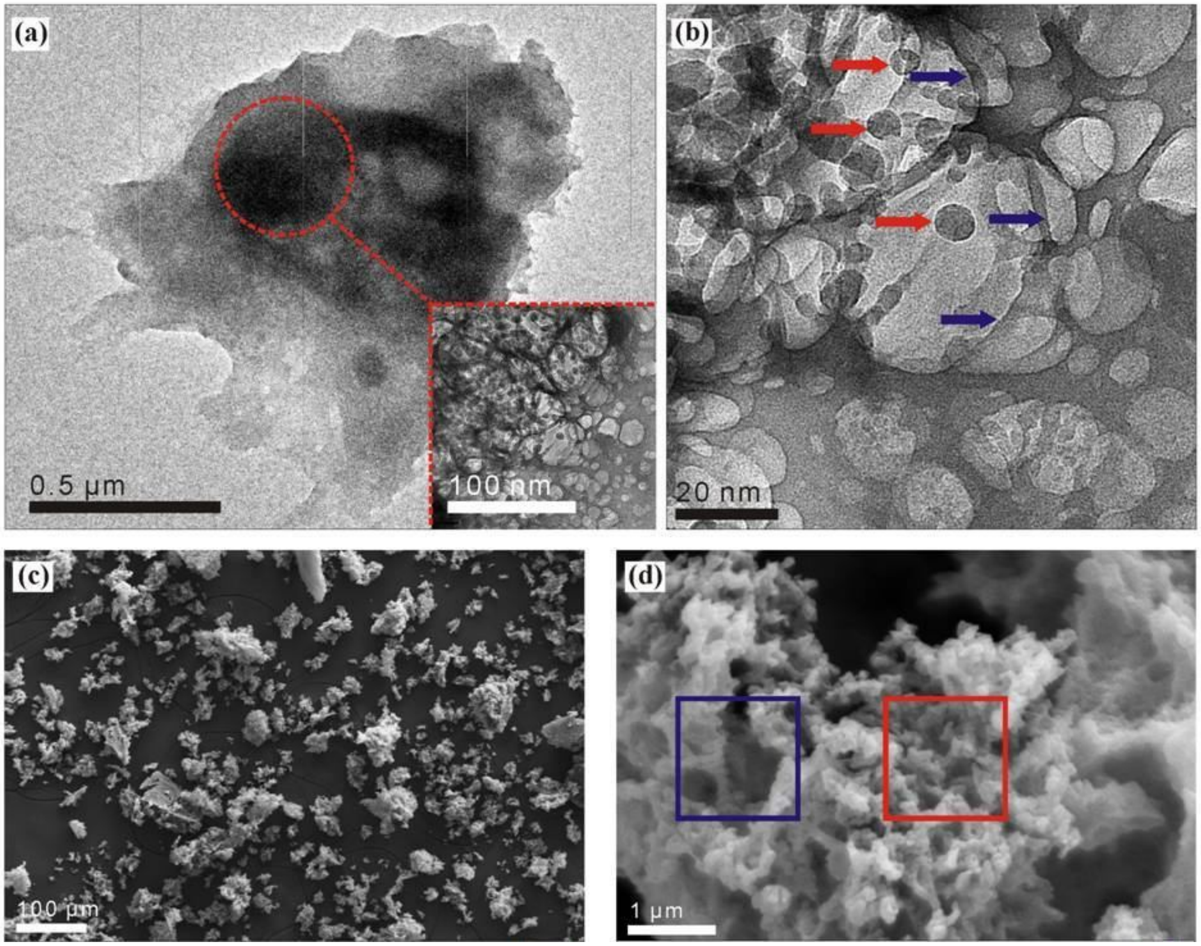

Figure S2. TEM micrographs of (a) aggregated Cu–chitosan NPs at 15kx, and at 42kx in inset showing porous network structures. (b) Cu embedded (red arrow) into chitosan porous network (blue arrow) at 110kx magnification. SEM images of (c) Cu–chitosan NPs at 230x, and (d) porous Cu–chitosan at 29.70 kx revealed nano (in red rectangular) and micro size pores (in blue rectangular). *The figure is adopted from reference (Saharan et al 2015)*[17] *with copyright permission from Elsevier.*

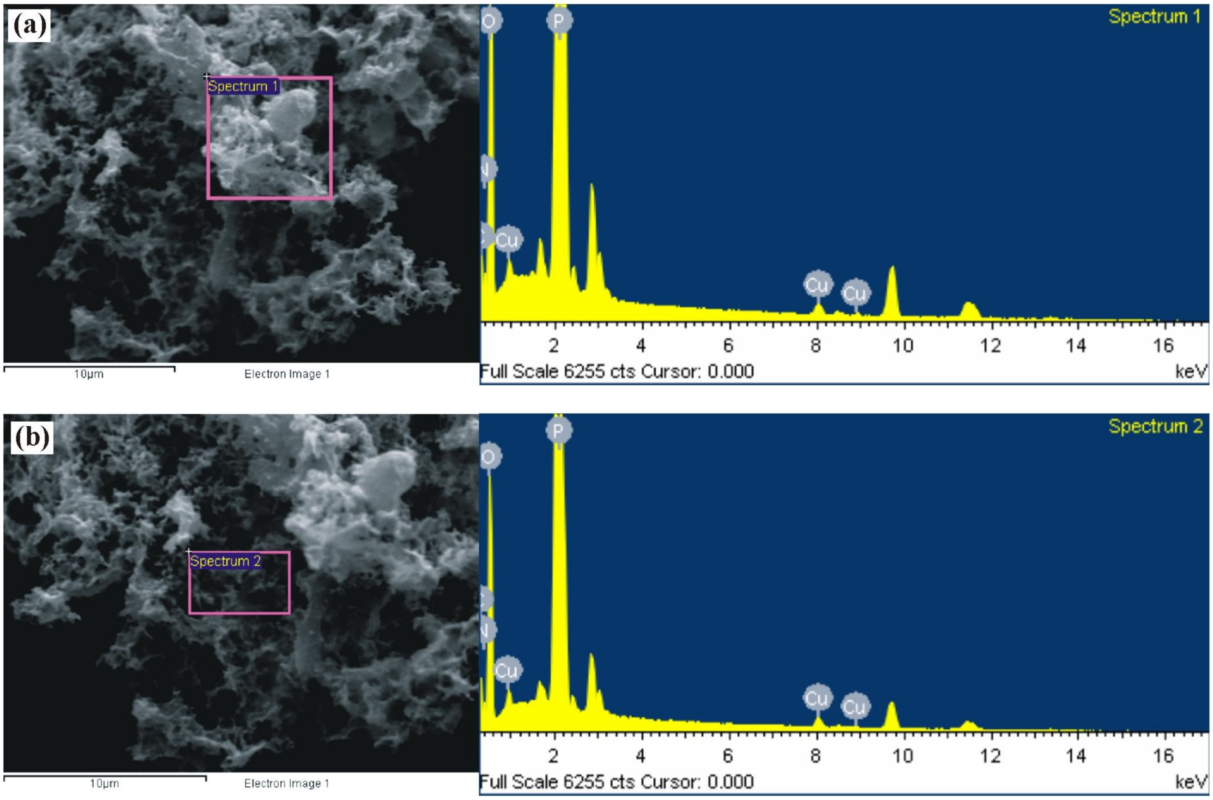

Figure S3. SEM-EDX elemental analysis of Cu–chitosan nanoparticles: (a) spectra of non-porous surface, and (b) spectra of porous surface. *The figure is adopted from reference (Saharan et al 2015)*[17] *with copyright permission from Elsevier.*

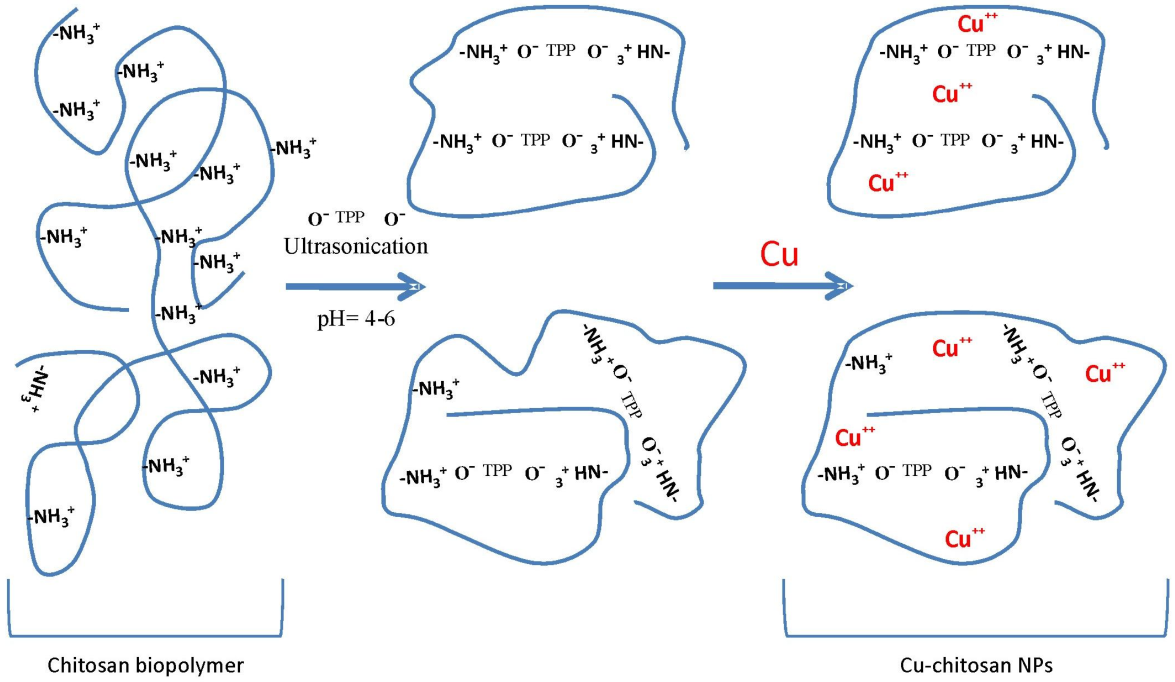

Figure S4. Hypothetical model of Cu-chitosan NPs. *The figure is adopted from reference (Saharan et al 2015)*[17] *with copyright permission from Elsevier.*
